# Effect of Beer Drinking

**Published:** 1890-03

**Authors:** 


### EFFECT OF BEER DRINKING.

The use of beer is found to produce a species of degeneration of all the organs. Profound and deceptive fatty deposits, diminished circulation, conditions of congestion, perversion of functional activities, and local inflammations of both liver and kidneys are constantly present. A stupor, amounting to almost paralysis, arrests the reason, changing all the higher faculties into a mere animalism, sensual, selfish, sluggish, varied only with paroxysms of anger that are senseless and brutal. In appearance the beer-drinker may be the picture of health, but in reality he is most incapable of resisting disease. Compared with inebriates who use different kinds of alcohol, he is more incurable and more generally diseased. The constant use of beer every day gives the system no recuperation, but steadily lowers the vital forces.

